# Indolent Angiomatoid Fibrous Histiocytoma Mimicking a Benign Cystic Tumor

**DOI:** 10.3390/diagnostics15010115

**Published:** 2025-01-06

**Authors:** Jiro Ichikawa, Tomonori Kawasaki, Kojiro Onohara, Masanori Wako, Satoshi Ochiai, Tetsuo Hagino, Hirotaka Haro

**Affiliations:** 1 Department of Orthopedic Surgery, Interdisciplinary Graduate School of Medicine, University of Yamanashi, Chuo 409-3898, Yamanashi, Japan; wako@yamanashi.ac.jp (M.W.); haro@yamanashi.ac.jp (H.H.); 2 Department of Pathology, Saitama Medical University International Medical Center, Hidaka 350-1298, Saitama, Japan; tomo.kawasaki.14@gmail.com; 3 Department of Radiology, Interdisciplinary Graduate School of Medicine, University of Yamanashi, Chuo 409-3898, Yamanashi, Japan; konohara@yamanashi.ac.jp; 4 Department of Orthopedic Surgery, National Hospital Organization (NHO) Kofu National Hospital, Kofu 400-8533, Yamanashi, Japan; hxcmk230@ybb.ne.jp (S.O.); tmhagino@amber.plala.or.jp (T.H.)

**Keywords:** indolent, angiomatoid fibrous histiocytoma, cystic lesion, imaging, histopathology, fluorescence in situ hybridization

## Abstract

Angiomatoid fibrous histiocytoma (AFH) is a rare intermediate tumor that is often difficult to diagnose radiologically and pathologically. Herein, we report a case of AFH in the knee that was initially misdiagnosed as a cystic lesion. The tumor was first identified eight years earlier during the patient’s initial visit, when plain magnetic resonance imaging (MRI) was performed, leading to a diagnosis of a cystic lesion. At the current visit, the tumor had subsequently enlarged, and pain had appeared. Contrast-enhanced MRI was performed at our hospital, revealing enhancement suggestive of a solid tumor. A needle biopsy was performed, raising suspicion of AFH. Wide resection was performed, and AFH was diagnosed using histopathological findings and fluorescence in situ hybridization (FISH). Although there are several characteristic imaging findings of AFH, they are non-specific, and small tumors can be easily overlooked. Furthermore, histopathological findings lack specific immunohistochemical markers, making morphological appearance, combined with FISH findings recently reported as useful, important for preventing misdiagnosis. Since cystic lesions can occur in various locations besides the knee, it is recommended to perform contrast-enhanced MRI for accurate diagnosis when there is an increase in size or the appearance of symptoms, as plain MRI alone may lead to misdiagnosis.

**Figure 1:**
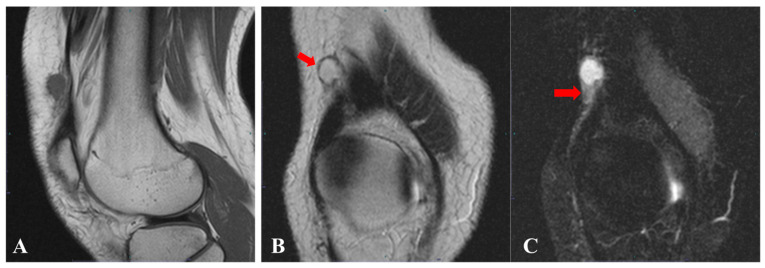
A 24-year-old female patient was referred to our hospital with a tumor in the proximal lateral aspect of the right knee. Eight years earlier, she had palpated a mass in the same location and visited a local clinic, where magnetic resonance imaging (MRI) was performed. MRI showed a tumor with iso-intensity signals on T1-weighted imaging (T1WI) compared with muscle, and high-intensity signals on T2-weighted imaging (T2WI) and fat-suppressed T2WI, measuring 12.5 × 10.5 × 9.5 mm (**A**). In addition, a pseudocapsule (**B**; red arrow) and peritumoral edema (**C**; red arrow) were identified. At that time, contrast-enhanced MRI was not performed. Based on these findings, a benign cystic tumor, such as ganglion or bursitis, was suspected, and careful follow-up was recommended.

**Figure 2:**
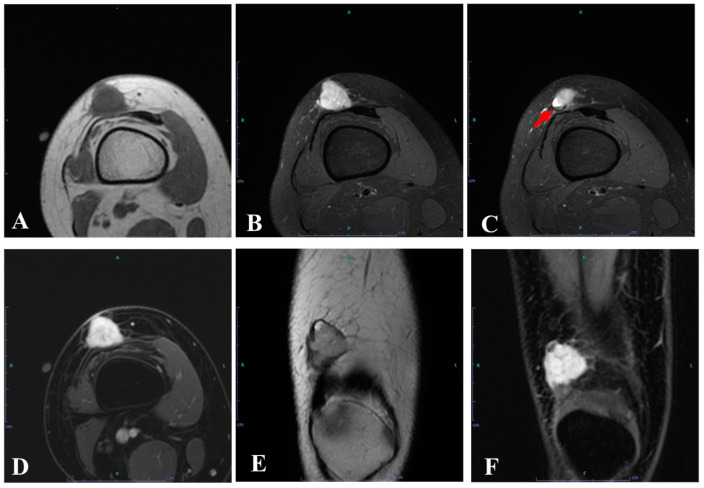
Over 8 years, the mass gradually increased in size, and the patient experienced occasional pain, prompting a return to the local clinic. Aspiration of the mass yielded no contents, suggesting that it was a solid lesion. The patient was then referred to our hospital for further evaluation. MRI at our hospital showed a tumor located subcutaneously adjacent to the vastus lateralis, with iso-intensity signals on T1WI compared with muscle (**A**) and heterogeneous high-intensity signals on T2WI (**E**) and fat-suppressed T2WI (**B**,**C**). The tumor was multiloculated, with a pseudocapsule (**E**), and contained a fluid level (**C**; red arrow) and peritumoral edema (**B**). It measured 22 × 18 × 30 mm. A heterogeneous enhancement effect was observed, excluding the cystic structure (**D**,**F**). An ultrasound-guided needle biopsy suggested angiomatoid fibrous histiocytoma (AFH). No metastasis was confirmed on contrast-enhanced CT. Characteristic MRI findings in AFH include a pseudocapsule, multilocular area, cystic area, fluid levels, and peritumoral edema [1,2]. However, since these findings can also be seen in various other soft tissue tumors, their specificity is low. Additionally, the sensitivity of these findings has not been evaluated in many cases, and cases without these findings have been reported [3], warranting further investigation. Contrast enhancement is generally seen in all cases, but enhancement patterns vary [1,2]. Considering that the average size of AFH tumors is generally small, there is a high possibility of overlooking the characteristic features, even with MRI. In our case, the characteristic findings were relatively easily identified on MRI at our hospital; however, even when analyzing the MRI from 8 years prior retrospectively after the diagnosis of AFH, several AFH features could be identified. It is extremely difficult to identify these features at a stage when the diagnosis has not yet been made. Differential diagnoses based on MRI include benign tumors (such as hemangioma and hematoma) and malignant tumors (such as synovial sarcoma and myxofibrosarcoma), making accurate diagnosis through imaging alone difficult [1]. Cystic tumors can occur in various locations, including the knee, and ganglions, bursitis, and atheroma are commonly encountered in daily practice. When determining whether a tumor is cystic, greater caution should be exercised when relying solely on plain MRI. In cystic tumors such as ganglions, there is no internal blood flow, giving contrast-enhanced MRI a significant advantage in differentiation. When there is an increase in tumor size or appearance of symptoms, contrast-enhanced MRI or at least Doppler ultrasound for blood flow evaluation should be performed.

**Figure 3:**
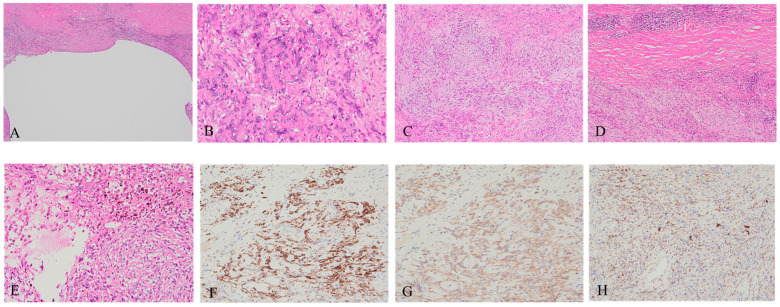
Considering the risk of recurrence, wide resection was performed (**A**) ×40, (**B**) ×200, (**C**) ×100, (**D**) ×100, (**E**) ×200, (**F**–**H**) ×200). Histopathologically, the tumor was composed of spindle cells with nuclear atypia (**B**) and minimal mitotic activity, along with cystic formation (**A**). The tumor was surrounded by a fibrous capsule with foci of lymphoplasmacytic cuffs (**D**). The spindle tumor cells showed perivascular whorled and storiform patterns with focal myxoid stroma (**C**), pseudoangiomatous spaces, and hemosiderin deposition (**E**). Immunohistochemistry (IHC) showed variable expression of desmin (**F**), CD99 (**G**), and CD68 (**H**), while MyoD1 (Figure S1A) and myogenin (Figure S1B) were negative. In diagnosing AFH, histopathological findings are the most important, with morphological findings being key due to the absence of specific IHC markers. The characteristic morphological features of AFH include (1) solid nodules of epithelioid to spindle cells with moderate eosinophilic cytoplasm and mildly atypical vesicular nuclei, (2) pseudoangiomatous spaces, (3) a thick fibrous pseudocapsule with hemosiderin deposition, and (4) a pericapsular rim of lymphoplasmacytic cells [4]. Notably, pseudoangiomatous spaces are only present in about two-thirds of cases, so caution is needed in diagnosis [5]. Additionally, in IHC, desmin is reported to have a high positivity rate, and EMA, CD99, and CD68 are also positive, but their positive rates varied across reports. Conversely, skeletal muscle markers, such as myogenin, MyoD1, S-100, and CD34, are generally negative [4,5]. The differential diagnosis encompasses a wide range of conditions, from benign to malignant [5,6], and there have been cases of misdiagnosis even after biopsy [3]. In particular, the diagnosis of the myxoid variant of AFH may be challenging.

**Figure 4:**
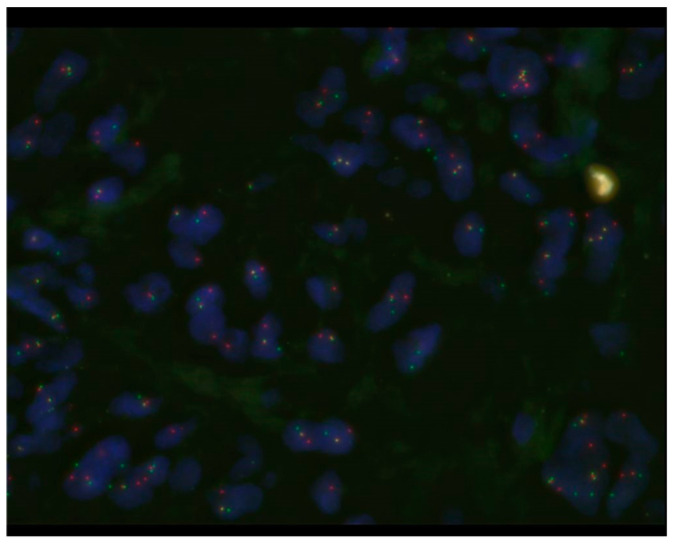
Fluorescence in situ hybridization (FISH) revealed EWSR1 split signals. Based on these pathological findings, we made the diagnosis of AFH. Recently, FISH has been reported to overcome diagnostic challenges [5,6]. AFH is characterized by three types of fusion genes: *EWSR1-CREB1*, *EWSR1-ATF1*, and *FUS-ATF1*, with the most common, *EWSR1-CREB1*, found in 90% of cases [5]. However, since *EWSR1-CREB1* and *EWSR1-ATF1* fusions are not exclusive to AFH, it is essential to combine FISH with histopathological findings for accurate diagnosis. Recurrence of AFH has been reported in about 10–30% of cases [1], and wide resection is recommended [5]. However, preoperative diagnosis is challenging, and whether additional wide resection should be performed after marginal resection remains unclear. Chemotherapy and radiation therapy have been used in metastatic and unresectable cases, but their effectiveness remains unclear [5]. Reported metastasis rates range from approximately 5% to 29%, and mortality rates similarly vary widely, from 0% to 14%, across reports, necessitating further studies with additional cases [1,4]. No specific histopathological factors have been identified as correlating with clinical outcomes [4].

## Data Availability

Not applicable.

## Supplementary Materials

The following supporting information can be downloaded at: https://www.mdpi.com/article/10.3390/diagnostics15010115/s1, Figure S1: Immunohistochemistry (IHC) showed MyoD1 (A ×200) and myogenin (B ×200) were negative.
