# Supplementary material for: Indolent Angiomatoid Fibrous Histiocytoma Mimicking a Benign Cystic Tumor
Source: Diagnostics (Basel). 2025 Jan 6;15(1):115. doi: 10.3390/diagnostics15010115 (PMC11720069; doi:10.3390/diagnostics15010115)
Supplement: Supplementary file 1 [file diagnostics-15-00115-s001.zip › diagnostics-3371185-supplementary material.pdf]

Supplementary Materials

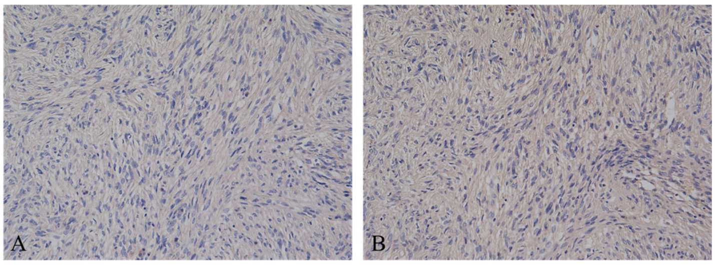

Figure S1 Immunohistochemistry (IHC) showed MyoD1 (A ×200) and myogenin (B ×200) were negative.
